# From ingestion to colonization: the influence of the host environment on regulation of the LEE encoded type III secretion system in enterohaemorrhagic *Escherichia coli*

**DOI:** 10.3389/fmicb.2015.00568

**Published:** 2015-06-05

**Authors:** James P. R. Connolly, B. Brett Finlay, Andrew J. Roe

**Affiliations:** ^1^Institute of Infection, Immunity and Inflammation, College of Medical, Veterinary and Life Sciences, University of Glasgow, Glasgow, UK; ^2^Michael Smith Laboratories, University of British Columbia, Vancouver, BC, Canada

**Keywords:** virulence, regulation, secretion, host, environmental, *Escherichia coli*

## Abstract

Enterohaemorrhagic *Escherichia coli* (EHEC) binds to host tissue and intimately attaches to intestinal cells using a dedicated type III secretion system (T3SS). This complex multi-protein organelle is encoded within a large pathogenicity island called the locus of enterocyte effacement (LEE), which is subject to extensive regulatory control. Over the past 15 years we have gained a wealth of knowledge concerning how the LEE is regulated transcriptionally by specific, global and phage encoded regulators. More recently, significant advances have been made in our understanding of how specific signals, including host or microbiota derived metabolic products and various nutrient sources, can affect how the LEE-encoded T3SS is regulated. In this review we discuss regulation of the LEE, focusing on how these physiologically relevant signals are sensed and how they affect the expression of this major virulence factor. The implications for understanding the disease process by specific regulatory mechanisms are also discussed.

## Introduction—Enterohaemorrhagic *E. coli* O157:H7

Enterohaemorrhagic *Escherichia coli* is a subset of the Shiga-toxigenic *E. coli* (STEC) group of pathogens. STEC are capable of causing disease that ranges from mild watery diarrhea to hemorrhagic colitis (HC) and in extreme scenarios hemolytic uremic syndrome (HUS; Nataro and Kaper, 1998; Croxen et al., 2013). HUS occurs as a result of Shiga-like toxin (Stx) exposure, a virulence factor carried by members of the STEC that is capable of ribosomal inhibition in susceptible cells. Subsets of STEC vary in their serotype and virulence factor reservoir with *E. coli* O157:H7 being the most extensively studied EHEC, and STEC for that matter, responsible for regular outbreaks of foodborne illness worldwide. Classically, O157:H7 infections have been associated with contaminated meat products but cases also appear in relation to contaminated fresh produce (Riley et al., 1983). Ruminants, most notably cattle, are the primary host for EHEC O157:H7, which they colonize asymptomatically at the recto-anal junction using the LEE-encoded T3SS (Karmali et al., 1983a,b; Orskov et al., 1987; Naylor et al., 2003). Cattle lack Gb3 receptors (the Stx receptor found on human endothelial cells) and are therefore not susceptible to Stx-associated pathogenesis (Pruimboom-Brees et al., 2000). Cattle provide a natural reservoir for EHEC O157:H7 and a direct link to subsequent infection of the human host.

The repertoire of virulence factors in EHEC O157:H7 includes a T3SS and its set of associated effector proteins, the Stx toxin as mentioned above and the pO157 plasmid, which have been reviewed extensively elsewhere (Croxen and Finlay, 2010; Croxen et al., 2013). Evidence for horizontal acquisition of virulence factors was highlighted in a study characterizing the complete genome sequence of the *E. coli* O157:H7 strain EDL933 (Perna et al., 2001). Genome sequence analysis revealed that EHEC contains hundreds of introgressed segments or “O-islands,” many of which carry virulence factors such as the LEE pathogenicity island (PAI). The study also characterized genomic regions related to known bacteriophage, including BP-933W encoding *stx_2_*, and a selection of cryptic prophage. These elements were found to encode various virulence related proteins such as T3SS effector proteins (Perna et al., 2001). Acquisition of horizontally acquired virulence factors can transform a harmless commensal into a niche specific pathogen. However, acquired virulence traits are not enough to confer pathogenicity alone, they must be adapted to the regulatory network of the cell for efficient expression in a temporal manner (Tan et al., 2015). Once appropriately integrated into the bacterial regulatory circuits these virulence factors can provide a competitive advantage for the emergent pathogen over the resident microbiota to thrive in this specific niche.

## The LEE Encoded Type III Secretion System

*Escherichia coli* O157:H7 forms attaching and effacing (A/E) lesions on host epithelial cells. This phenotype is characterized by intimate attachment to the host cell surface, subversion of the host actin cytoskeleton and raised “pedestal” formation (Moon et al., 1983; McDaniel et al., 1995), a hallmark of O157:H7 pathogenesis. Similarly, enteropathogenic *E. coli* (EPEC) and the mouse pathogen *Citrobacter rodentium* also confer the A/E phenotype on host cells (Moon et al., 1983; Schauer and Falkow, 1993; Croxen et al., 2013; Collins et al., 2014). The ability to form A/E lesions has been attributed to the carriage of the ∼35 kb LEE PAI, which contains all the necessary genes for the formation of a functional T3SS (McDaniel et al., 1995; McDaniel and Kaper, 1997; Wong et al., 2011). The LEE has a GC content of 38.3%, which is in contrast to the 50.8% that constitutes the *E. coli* chromosome, suggesting that the LEE was acquired by horizontal gene transfer (McDaniel et al., 1995; McDaniel and Kaper, 1997; Elliott et al., 1998). Indeed, whole contribution of the LEE to the A/E phenotype was confirmed when the entire LEE from the EPEC strain E2348/69 was introduced to the non-pathogenic K-12 strain resulting in the ability to form A/E lesions (McDaniel and Kaper, 1997). In contrast, the EHEC LEE is incapable of conferring virulence on K-12 suggesting that LEE adapts to different genetic backgrounds in unique ways (Elliott et al., 1999).

The LEE contains 41 open reading frames (ORFs) that include genes encoding the T3SS basal apparatus and secretion machinery, the translocon needle filament subunit EspA (Knutton et al., 1998), the translocon pore proteins EspD and EspB (Ide et al., 2001), the major adhesin intimin Eae and its cognate “translocated intimin receptor” Tir (Kenny et al., 1997b), a lytic transglycosylase EtgA to clear peptidoglycan localized around the forming T3SS (Burkinshaw et al., 2015), a selection of secreted effector proteins and cellular chaperones (reviewed, in Frankel et al., 1998 and Wong et al., 2011). As well as LEE encoded effector proteins this T3SS is also capable of translocating a large number of non-LEE encoded effectors, located throughout the chromosome on horizontally acquired genetic elements, that are important for the virulence process (Tobe et al., 2006; Deng et al., 2012). The 41 ORFs can be largely organized into five polycistronic operons, annotated LEE1 through LEE5 with LEE1, LEE2, and LEE3 encoding the major structural components of the T3SS, LEE4 encoding the translocon pore and LEE5 encoding Tir and Intimin (Elliott et al., 1998). ORF 1 on the LEE1 operon encodes the master regulator Ler (LEE encoded regulator) through which activation of operons LEE2 to LEE5 is mediated (Mellies et al., 1999; Sánchez-SanMartín et al., 2001; Haack et al., 2003). A second master regulatory system, GrlRA, is located between the LEE1 and LEE2 operons, is activated by Ler and forms a regulatory feedback loop on LEE1 expression (Deng et al., 2004). Thus, the LEE contains all the necessary equipment to form a functional T3SS, the structure of which has been recently reviewed in detail (Büttner, 2012), capable of mediating the A/E phenotype.

Expression of the LEE is tightly regulated in response to multiple stimuli. Host temperature of 37°C provides optimal LEE expression as well as the exponential growth phase (Rosenshine et al., 1996). Experimentally, the LEE can be induced by growth in tissue culture media (DMEM or MEM-HEPES) as these conditions mimic the physiological environment (Roe et al., 2003). A detailed study by Kenny et al. (1997a) attributed *in vitro* secretion to no single component but rather a combination of temperature, pH, osmolarity, calcium, iron and salt concentrations.

Transcriptional regulation of the LEE is extremely complex. It is controlled at the core level by two integral regulatory systems encoded within the LEE, Ler and GrlRA (Deng et al., 2004). Numerous other systems such as nucleoid regulators, stress response regulators and various environmental sensing systems make contributions to regulation of the LEE. Chromosomal and phage encoded genetic elements also feed into the LEE creating layers of specific control on the system. Mellies et al. (2007) have previously reviewed regulation of the LEE in great detail for EHEC and EPEC. In this review, we summarize the key points of LEE regulation in relation to EHEC primarily whilst also commenting on EPEC and *C. rodentium* where appropriate. We provide an up to date summary of the regulatory systems involved in LEE control and later focus in more detail on the specific responses of this PAI to various physiologically relevant stimuli that EHEC can encounter during colonization of the bovine or human hosts. It is important to emphasize that LEE regulation has both common elements and distinct differences between the pathotypes of EHEC, EPEC or *C. rodentium* that will be mentioned in this review, and this can be attributed to the different environments encountered within the host that they primarily colonize.

## Master Regulation of the LEE

Transcription of the LEE is orchestrated largely by the ∼15 kDa protein Ler that activates transcription at each of the subsequent LEE operons as well as non-operonic members of the LEE such as *map*, *espG*, and *escD* (Mellies et al., 1999; Elliott et al., 2000; Sánchez-SanMartín et al., 2001; Haack et al., 2003). The importance of Ler for virulence was demonstrated by way of a deletion mutant in EHEC strain 86-24. This mutant was impaired in its ability to secrete effectors and form A/E lesions on host cells. Additionally, the *ler* mutant strain showed a decreased expression of non-LEE encoded virulence factors by western blot (Elliott et al., 2000). StcE, a non-T3SS secreted metalloprotease encoded on the pO157 plasmid, was demonstrated to be under the control of Ler, implying a global control of virulence gene regulation by Ler (Lathem et al., 2002). Additionally, a number of non-LEE encoded effector proteins that are translocated into the host cell via the T3SS are also under direct control by Ler (Roe et al., 2007; Holmes et al., 2012). A comprehensive study by Deng et al. (2004) systematically analyzed the role of each ORF in virulence by deletion within the LEE of *C. rodentium.* This work further highlighted the absolute importance of Ler for virulence by the T3SS in a mouse model of infection, thus expanding the knowledge of LEE regulation to other pathogens (Deng et al., 2004).

Transcriptional regulation of *ler* specifically is complex. The LEE1 operon contains an unusually long upstream leader sequence of ∼170 bases (Islam et al., 2012). In EHEC, EPEC and *C. rodentium* transcription is driven through a promoter designated P1 (distal) however, in EHEC transcription has been demonstrated to be driven from not one but two promoters, designated P1 and P2 (proximal), as well as a cryptic promoter (P1A) that overlaps P1 (Sperandio et al., 2002a; Islam et al., 2011b). The precise contributions of each promoter are unclear but investigations into the activity of the LEE1 promoter region has identified that P1 is likely to be the major promoter of the LEE1 operon in EHEC with the P2 promoter being active to a lesser extent (Islam et al., 2011a). Ler has also been demonstrated to elicit negative autoregulation on the LEE1 promoter as well as positively drive expression from operons LEE1 through LEE5, presumably as a mechanism of optimizing LEE expression levels to steady-state during the infection process (Berdichevsky et al., 2005).

## The GrlRA Regulatory Feedback Loop

**FIGURE 1:**
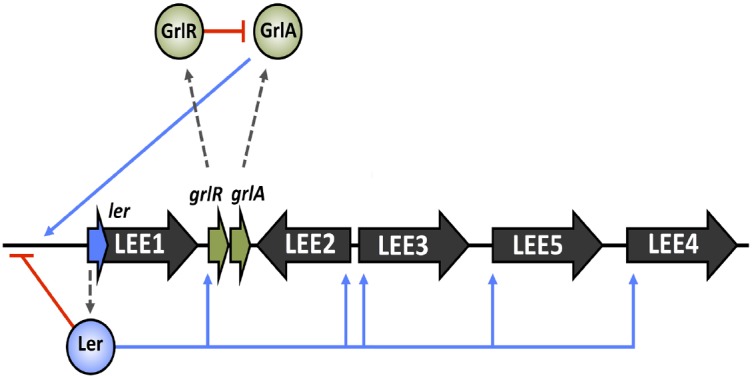
**Core regulation of the LEE island.** LEE operons 1–5 are indicated in black with the *ler* and *grlRA* ORFs highlighted in blue and green respectively. Broken gray arrows correspond to translated products from these genes. Blue lines represent input points of positive transcriptional regulation whereas red blunt arrows indicate negative regulation.

The analysis of systematic LEE mutations by Deng et al. (2004) revealed a second LEE-encoded regulatory system that was indispensable for the LEE function. The work identified a positive regulator of *ler* expression, named GrlA (global regulator of *ler* activation), and a negative repressor of *ler*, GrlR (global regulator of *ler* repression; Deng et al., 2004). GrlA forms a positive feedback loop with Ler to maintain steady activation of the LEE under positive conditions, whereas GrlR was postulated to inhibit GrlA mediated activation of *ler* in order to tightly control LEE expression (Barba et al., 2005). Recently the structure of GrlR and a GrlR/GrlA complex has been solved and a mechanism of Ler regulation by this system proposed. GrlR forms a dimeric structure in solution that binds GrlA, stabilizes it and inhibits transcriptional activation of the LEE1 operon (Padavannil et al., 2013). This complements other findings that suggest under certain conditions, such as those favoring LEE expression, GrlR is cleaved by the ClpXP protease, freeing GrlA and allowing *ler* transcriptional activation (Iyoda and Watanabe, 2005). Despite these findings, the precise mechanism governing GrlR antirepression is unknown. An overview of LEE master regulation is illustrated in Figure 1.

## Regulation of the LEE by Bacterial Nucleoid Associated Proteins

Nucleoid-associated proteins (NAPs) are found across all lineages of life (Eukarya, Bacteria, and Archaea) and play global roles in nucleoid maintenance. The DNA-binding and shaping ability of NAPs allows them to affect more than just chromosome architecture and they have emerged as global regulators of transcription (Dillon and Dorman, 2010). Perhaps of most relevance to the LEE is the histone-like nucleoid structuring protein (H-NS). This regulator forms DNA-bridges over extended stretches of DNA in the vicinity of promoters under its control, thus blocking or trapping DNA polymerase and “silencing” the promoter (Dame et al., 2006; Grainger et al., 2006; Dorman, 2007). H-NS silencing is biased toward foreign AT rich segments of the DNA and is thought of as a global repressor of horizontally acquired genetic elements (Navarre et al., 2006; Oshima et al., 2006). Overcoming H-NS repression is often an adaptation of pre-existing global regulators but can occur via horizontally acquired elements also, as is the case for Ler (Abe et al., 2008). The LEE is repressed by H-NS under non-inducing conditions such as low temperature. Ler acts as an H-NS antagonist by displacement from the LEE thus relieving H-NS mediated silencing (Umanski et al., 2002).

**FIGURE 2:**
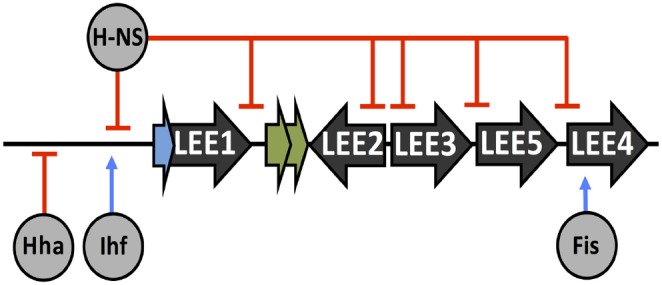
**Regulation of the LEE by nucleoid associated proteins (NAPs).** Points of transcriptional activation (blue arrow) or repression (red blunt arrow) by NAPs, which are labeled and indicated in gray.

As well as H-NS, other NAPs have been reported to contribute to LEE regulation. Integration host factor (Ihf) is a dimeric NAP composed of alpha and beta subunits. Ihf is capable of wrapping DNA and affecting transcription on a genome-wide scale. A global DNA binding study of Ihf in *E. coli* K-12 revealed that Ihf was capable of binding with sequence-specificity to ∼30% of all operons in *E. coli* K-12, however, not all of these operons were transcriptionally affected in an Ihf mutant background suggesting accessory regulation (Dillon and Dorman, 2010; Prieto et al., 2012). Ihf positively promotes *ler* expression by binding upstream of the LEE1 promoter, aiding H-NS displacement and promoting full LEE expression (Friedberg et al., 1999). In contrast, the NAP Hha represses *ler* transcription by a proposed DNA binding mimicry mechanism, concentrating H-NS repression on the LEE (Sharma and Zuerner, 2004; Madrid et al., 2007). The factor for inversion stimulation (Fis) is responsible for early exponential phase transcriptional regulation and has been demonstrated to affect *ler* and LEE4 expression in EPEC (Goldberg et al., 2001). Collectively, the cellular NAPs have been adapted by the cell maintain control over the LEE. NAP input to the LEE is illustrated in Figure 2.

## Cross-talk between Genetic Regulatory Elements and the LEE

**FIGURE 3:**
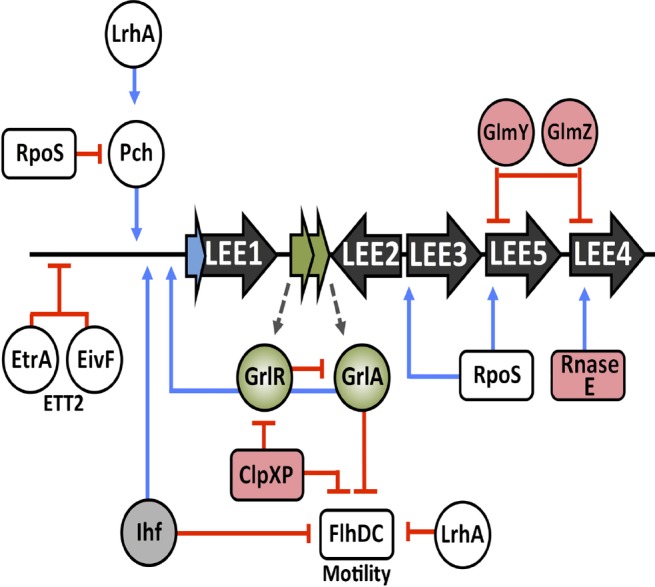
**Transcriptional cross-talk of cellular regulators and post-transcriptional control of the LEE.** Inputs from various transcriptional regulators, indicated in white, and the cross-regulation associated with them. Regulators of the ETT2 system and motility are labeled accordingly. Post-transcriptional regulators are shaded in pink, Ihf is shaded in gray and GrlRA are shaded in green. Blue lines represent positive regulation whereas red blunt arrows indicate negative regulation.

The LEE, like many horizontally acquired virulence factors, must be regulated appropriately to ensure efficient expression in a timely manner. There are a number of global regulators, both chromosomally encoded and horizontally acquired that contribute to LEE regulation (illustrated in Figure 3). In EPEC, the *perABC* family of AraC-like transcriptional regulators are encoded on the EAF virulence plasmid but this system is not found in EHEC (Mellies et al., 1999). Instead, three functional homologs of the direct EPEC Ler activator PerC, *pchABC* encoded on lambdoid prophages, were identified in O157:H7 and found to be required for full virulence (Iyoda and Watanabe, 2004). Expression of these Pch regulators was required for full LEE activation during growth in tissue culture medium (LEE-inducing) or interaction with host cells. Furthermore, Abe et al. (2008) comprehensively demonstrated the binding capacity of Pch to LEE associated ORFs, non-LEE associated effectors and a variety of other global sites including those involved in acid resistance and NAPs.

Cross-regulation between the LEE and flagella is an intriguing concept. Classically, EHEC are known to be non-motile under LEE-inducing conditions *in vitro* (Tobe et al., 2011). The H7 flagellum has adhesive properties to the bovine epithelium, mediating initial attachment to host cells in the early stages of infection followed by a regulatory switch in LEE activation and subsequent intimate attachment via the T3SS (Mahajan et al., 2009). As discussed above, GrlR repression of GrlA is removed by the ClpXP protease, thus promoting LEE expression by GrlA (Padavannil et al., 2013). Furthermore, ClpXP is capable of post-translationally inactivating the flagellar master regulator, FlhDC (Kitagawa et al., 2011). Additionally, GrlA transcriptionally represses *flhDC* resulting in two independent mechanisms of motility inhibition under LEE inducing conditions (Iyoda et al., 2006). Ihf, which is required for LEE1 expression has also been shown mediate *flhDC* repression (Friedberg et al., 1999; Yona-Nadler et al., 2003). LrhA is a LysR type transcriptional regulator known to repress motility in a K-12 background. LrhA is important for LEE1 activation by directly activating PchA and PchB (Lehnen et al., 2002; Honda et al., 2009).

Genome sequencing of O157:H7 revealed the presence of a second cryptic Type III secretion system designated ETT2 (Hayashi et al., 2001; Perna et al., 2001). Functionality and the role of the ETT2 system is controversial with the locus being subject to widespread mutational attrition (Ren et al., 2004). However, two regulators encoded by the system, EtrA and EivF, were found to be functional repressors of the LEE in EHEC. This study highlighted the cross-talk between two independently acquired PAI and was hypothesized to mediate avoiding the expression of two T3SSs simultaneously. The work also demonstrates the ability of remaining genes from degenerate systems to have residual effects on the cell and play roles in regulation (Zhang et al., 2004).

Hfq, the small RNA (sRNA) chaperone, is a global post-transcriptional regulator of gene expression. The LEE was found to be negatively regulated through Hfq-mediated modulation of the *ler* and *grlRA* mRNA transcripts (Hansen and Kaper, 2009; Shakhnovich et al., 2009). Conversely, in strain 86-24, Hfq was attributed to an indirect activation of the LEE in this genetic background (Kendall et al., 2011). Strain to strain variation in LEE expression by global regulators has also been documented in relation to the stationary phase sigma factor RpoS. In 86-24 LEE3 and *tir* were found to be dependent on RpoS (Sperandio et al., 1999). This is in contrast to studies using the Sakai O157:H7 strain that reported repression of the LEE by RpoS (Iyoda and Watanabe, 2005). Global gene expression profiling of an *rpoS* mutant in EDL933 revealed a dependency of *ler* expression on RpoS but many LEE encoded ORFs were unaffected, whereas analysis of an *rpoS* mutant in the mouse pathogen *C. rodentium* revealed a positive influence of RpoS on LEE-mediated infection *in vivo* (Dong and Schellhorn, 2009; Dong et al., 2009). Although, the role of RpoS in LEE regulation is influenced by growth conditions these studies collectively demonstrate strain-to-strain variation in virulence factor expression depending on the genetic background examined.

Post-transcriptional regulation of the LEE is a less explored but relevant topic. As mentioned, Hfq has been reported to regulate the LEE in a strain-specific manner. A number of studies have detailed the role of small regulatory RNAs (sRNA) in control of LEE expression. The RpoS stimulatory sRNA, DsrA, stimulates the expression of *ler* in an RpoS dependent manner (Laaberki et al., 2006). Recently, two sRNAs involved in activation of glucosamine biosynthesis (GlmY and GlmZ) were found to play global roles in gene expression that included post-transcriptional regulation of the LEE4 and LEE5 operons as well as the non-LEE encoded effectors NleA and EspFu providing further evidence of the complexity of LEE control (Gruber and Sperandio, 2014, 2015). Transcription from the LEE4 operon, encoding a combination of T3SS components and regulators has also been demonstrated to be under post-transcriptional modification by the cellular endonuclease Rnase E allowing specific regulation of protein production from these transcripts (Lodato and Kaper, 2009).

## The Adaptable GAD Regulators and the Response to Nitric Oxide

Foodborne pathogens must survive ingestion and passage through the stomach to reach their intestinal niche. *E. coli* employs specific mechanisms to counteract the harsh acidic environment of the stomach, such as the glutamate-decarboxylase (GAD) acid stress response (De Biase et al., 1999). The GAD system is regulated globally by multiple factors including H-NS, PchA, PchB, and RpoS (Atlung and Ingmer, 1997; De Biase et al., 1999; Giangrossi et al., 2005). The system is a complex signaling cascade that ultimately senses environmental pH and responds accordingly by influencing transcription of the acid tolerance gene network (Masuda and Church, 2003).

Aside from regulating acid resistance, transcriptional regulators of the GAD system have shown adaptability to transcriptional control of the LEE. GadX has been demonstrated to directly repress *perA* in EPEC, thus inhibiting PerC activation of the LEE (Shin et al., 2001). In EHEC, a transposon mutant screen identified GadE and YhiF mutants that displayed increased activity at the LEE2 and LEE4 operons by a mechanism independent of *ler*. The mutants were increased in their ability to colonize host cells and infect mice (Tatsuno et al., 2003). As discussed above, both horizontally acquired and chromosomally encoded regulators can have direct and indirect effects on LEE expression. Tree et al. (2011) described the prophage-encoded secretion regulators (Psr) found on numerous O-islands in O157:H7. The mechanism of regulation involved GadE and YhiF mediated Psr repression of the LEE, largely through repression of the LEE2, LEE3, and LEE5 operons. Furthermore, the study demonstrated direct binding of purified GadE to the LEE1 and LEE2/3 promoters indicating both direct and indirect control over LEE expression. It was proposed that the Psr system has been adapted to promote the expression of prophage-encoded effectors that employ the T3SS for translocation into the host cell. This is achieved by repressing LEE-encoded effectors at later time points during colonization (Tree et al., 2011). Based on these reports it was hypothesized that the GAD regulators have adapted to control the LEE as well as the acid stress regulon. The reason for this adaptation is likely to inhibit the expression of colonization factors under conditions of stress, such as the acidic environment of the stomach. Once enteric pathogens have passed through the stomach and into the intestine, this more favorable environment allows lifting of GAD repression on the LEE. The latter study demonstrates not only the ability of the GAD system to regulate the LEE in response to the environment but also demonstrate further how horizontally acquired regulators such as Psr can override pre-existing regulatory networks to mediate regulation of virulence factors in efficient ways.

**FIGURE 4:**
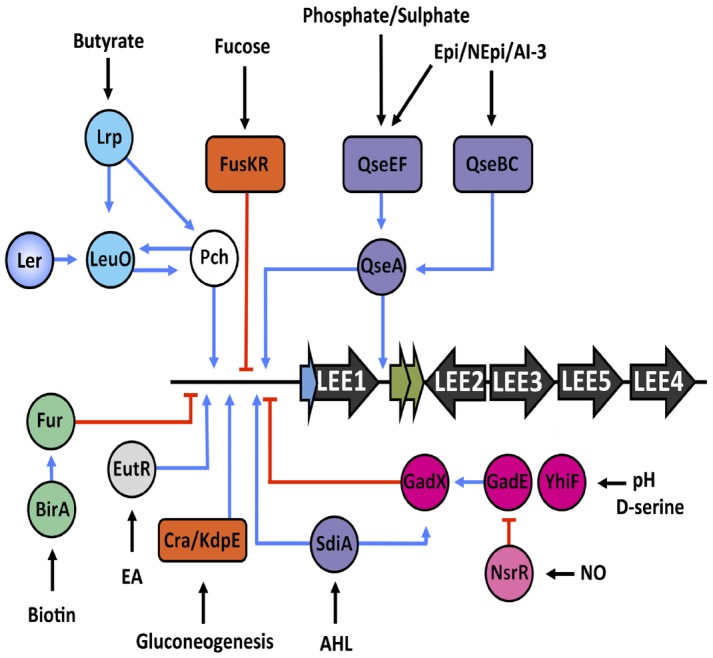
**Regulatory map of various signals encountered by EHEC within the host.** Black arrows indicate the signal being sensed and the regulatory systems are color coded as follows—quorum sensing of hormones and hormone-like signals (purple), nutrient sensing (orange), nitric oxide sensing (pink), pH and D-serine regulation (magenta), ethanolamine sensing (light gray), butyrate sensing (aqua), and biotin sensing (green). Blue lines represent input points of positive transcriptional regulation whereas red blunt arrows indicate negative regulation.

Nitric oxide (NO) is a key mediator of the host innate immune response (Foley and O’Farrell, 2003). Recently, GadX and GadE were implicated in NO mediated repression of the LEE through a complex regulatory interplay with the nitrite sensitive repressor NsrR. The work described how under LEE-inducing conditions GadE, and therefore GadX, is repressed and NsrR activates the LEE. In this context, NO exposure lifts NsrR repression of GadE and results in activation of LEE1 and LEE4/LEE5 by GadX and GadE respectively. In a manner similar to the GAD system, the LEE is therefore repressed in the stomach due to the abundance of NO found in gastric juices and the GAD regulators are recruited to enhance this response (Branchu et al., 2014). These studies highlight the adaptive power of the GAD regulators to mediate not only the response to acid stress but also temporal expression of the LEE within the host (Figure 4).

## Chemical Signaling in a Complex Environment—LEE Regulation by Quorum Sensing

Interspecies communication by way of quorum sensing (QS) involves the production of hormone-like signals by bacterial populations that are subsequently sensed and perceived by two-component regulatory systems (TCS). These systems consists of a surface exposed Histidine-kinase sensor protein that autophosphorylates in response to certain stimuli. This phosphate is subsequently transferred to a cellular response regulator and the signals are transmitted to the genome for differential regulation of signal-sensitive genes (Hughes and Sperandio, 2008). QS however, has evolved to allow the sensing of not only bacterially derived signals, such as the auto-inducer 3 signaling molecule (AI-3), but also host-derived hormones, namely the stress hormones epinephrine (Epi) and norepinephrine (NEpi), in a synergistic manner (Walters and Sperandio, 2006). Sensing of these host-derived cues provides EHEC with important inputs into the physiology of the host and resident microbiota. Two TCSs (QseBC and QseEF) involved in QS have been described in detail and play key roles in regulation of virulence and motility in EHEC and signals are transmitted through two LysR type transcriptional regulators, QseA and QseD (Sperandio et al., 1999, 2002b; Russell et al., 2007; Sharp and Sperandio, 2007; Habdas et al., 2010). QseA can both directly and indirectly activate expression of the LEE1 operon by binding to both the P1 and P2 promoter regions. Additionally, QseA has been demonstrated to directly activate expression of O-islands encoding non-LEE effectors (Kendall et al., 2010). Epi, NEpi and AI-3 feed in via the sensor kinase QseC, thus phosphorylating the response regulator QseB (Clarke et al., 2006). QseB in turn positively regulates motility through direct interaction with the *flhDC* regulatory region (Clarke and Sperandio, 2005). QseEF can sense Epi, phosphate and sulfate then signal to QseA in a QseC dependent manner (Reading et al., 2007, 2009).

Enterohaemorrhagic *Escherichia coli* can also sense hormone-like signals produced by members of the microbiota. SdiA is a LuxR-like transcriptional regulator has the remarkable ability of responding to acyl-homoserine lactones produced by neighboring bacteria but not EHEC (Hughes et al., 2010; Nguyen et al., 2013). SdiA represses the LEE in response to these signals but also activates expression of GadX known to inhibit LEE1 expression as EHEC passage through the stomach. The rumen of cattle is very abundant in acyl-homoserine lactones thus limiting colonization of EHEC to more distal sites of the gastrointestinal tract, such as the recto-anal junction (Kanamaru et al., 2000; Naylor et al., 2003; Hughes et al., 2010).

## Competition for Nutrients and the Expression of Virulence

The intestine is a complex environment where commensals have adapted to a specific niche with specific nutrients. Incoming pathogens are therefore unwanted visitors and are in direct competition for the limited nutrient supply of this environment. Chang et al. (2004) have described by systematic mutagenesis of metabolic pathways that only mutation in sugar utilization pathways affected competitive colonization of *E. coli* in a mouse model. Gluconate was found to be the primary carbon source for K-12 with glucuronate, mannose, fucose and ribose contributing to niche maintenance (Chang et al., 2004). Utilization of unique sugar sources therefore allows pathogenic *E. coli* to establish distinct niches within this competitive environment. The researchers have recently proposed “The Restaurant Hypothesis” which states that inhabitants of mixed biofilm populations can locally dictate which nutrients are available (Leatham-Jensen et al., 2012; Meador et al., 2014). In order to overcome competition for limited nutrients, EHEC utilizes both glycolytic and gluconeogenic carbon sources to temporally regulate initial colonization and long term establishment respectively (Miranda et al., 2004). Expression of *ler* is directly inhibited by glycolytic by-products and promoted by gluconeogenic conditions respectively by the catabolite/osmotic stress responsive Cra/KdpE system (Njoroge et al., 2012). The binding capacity of Cra and KdpE to the LEE1 regulatory region is dependent on signal transduction the Histidine kinase KdpD, with the phosphorylation state of KdpE being determined by the concentration of glucose in the environment.

In a study investigating the transcriptomic profiles of EHEC persistence in the bovine small intestine, the importance of gluconeogenic metabolism was assessed. The study found regulons involved in ethanolamine (EA), urea and amino acid utilization were all upregulated under these *in vivo* conditions (Bertin et al., 2014). This study agreed with an earlier observation that EA, a cell membrane component released into the gut lumen, provided EHEC with a competitive advantage over the resident microbiota, which normally do not metabolize EA (Bertin et al., 2011). More recently, EA was confirmed as a signal for EutR, the transcriptional regulator, which drives expression of the LEE1 operon and EA metabolic genes independently of one another (Kendall et al., 2012; Luzader et al., 2013). EA has also been attributed to activation of putative O157:H7 fimbrial-like loci, supporting the theory that EA plays a physiologically relevant role in EHEC colonization (Gonyar and Kendall, 2014).

Fucose is a sugar found in abundance (∼100 μM) in the human or bovine intestine and is one of the preferred carbon sources utilized by *E. coli* for maintenance of intestinal colonization (Chang et al., 2004). Fucose is derived from host glycans by the gut commensal *Bacteroides thetaiotaomicron*. EHEC utilizes the FusKR TCS to avoid competition with resident *E. coli* for this carbon source. This system has been recently acquired in the evolutionary timeline and is regulated in response to the presence of epithelial mucus. The system acts to directly repress the LEE1 operon and indirectly repress fucose utilization genes in response to high concentrations of fucose available in the intestinal mucus layer, thus limiting the possibility of unnecessary competition for this carbon source. Once in close contact with the intestinal epithelium, a niche devoid of commensal *E. coli*, QS systems activate LEE1 expression and lift fucose mediated repression of the LEE, therefore allowing establishment of A/E associated colonization of the host epithelium (Pacheco et al., 2012).

These studies provide elegant examples of how nutrient metabolism and virulence must be co-regulated to work together but not dependently on each other in order to carefully control niche-specific virulence in response to particular environmental nutritional content. A summary of environmental signals that feed into the regulation of the LEE is found in Figure 4.

## A Link between Virulence and Metabolism—Regulation by Short Chain Fatty Acids and Biotin in the Host Intestinal Tract

As well as carbon and nitrogen sources, the intestine is abundant in short chain fatty acids (SCFAs) such as butyrate, acetate and propionate that can reach concentrations of up to 140 mM (Cummings and Macfarlane, 1991). SCFAs are end products of bacterial fermentation of dietary carbohydrates such as resistant starches or fiber and are thought to provide a nutrient source for maintenance of a healthy colon of both humans and animals (Wong et al., 2006). Studies have also shown that SCFAs play a distinct role in LEE regulation being adapted as niche-specific signals helping to trigger colonization of EHEC. Nakanishi et al. (2009) showed that butyrate could significantly increase LEE expression in a PchA dependent manner at concentrations that were not inhibitory to growth. This regulation required another regulator, the leucine-responsive protein (Lrp), which is a global transcription factor necessary for nitrogen metabolism in *E. coli* (Nakanishi et al., 2009). Lrp has been shown in K-12 to act at 138 different binding sites including that of *lrhA* (Cho et al., 2008). Thus, butyrate activation of the LEE may be via an Lrp-LrhA-PchA signaling cascade. Recently, Takao et al identified the global LysR type transcriptional regulator LeuO as a mediator in the Lrp dependent system. They showed that butyrate induced *leuO* and *pchA* via Lrp, subsequently activating *ler* via PchA. Ler and PchA were capable of directly activating *leuO* suggesting the system forms a positive feedback loop upon *leuO* induction (Takao et al., 2014).

Short chain fatty acids not only regulate virulence associated with the LEE but also motility. Addition of SCFAs to growth in DMEM, conditions promoting LEE expression that are normally repressive of motility, activated motility in EHEC. Butyrate was capable of simultaneously activating motility and LEE expression in an Lrp dependent manner. This occurred by direct activation of the *flhDC* flagellar master regulators. Acetate and propionate on the other hand could not induce LEE expression and induced motility further down the regulatory hierarchy. Due to the known adhesive properties of the H7 flagellum these findings further bolster the proposed role of flagella in initial attachment to the host epithelia (Tobe et al., 2011). In a study characterizing the protein targets of a class of type 3 secretion inhibitors, the salicylidene acylhydrazides (SA), a number of putative cellular targets were implicated in regulation of virulence (Wang et al., 2011). One of these was AdhE, an alcohol dehydrogenase. Deletion of *adhE* resulted in a pleiotropic phenotype—overproduction of extracellular acetate, expression of non-functional flagella and post-transcriptional inhibition of the T3SS. The findings highlight the fine balance required between central metabolism and regulation of virulence factors in order to appropriately function in the environment. It is known that cattle contain high concentrations of SCFAs particularly in the rumen. It is therefore conceivable that lower concentrations of acetate in the rectum, the major colonization site for EHEC in cattle, would promote T3SS mediated attachment more efficiently (Beckham et al., 2014).

Similarly to SCFAs, the distribution of the essential cofactor biotin within the intestine has recently been proposed to be an important determinant of EHEC niche specification. Yang et al. (2015) revealed that biotin concentrations in the small intestine were 14-fold higher than that of the large intestine, the preferred colonization site of EHEC, and that this correlated with a 31.5-fold higher EHEC bacterial count associated with the large intestine during infection of mice. The study proposed a mechanism of niche specification whereby the biotin protein ligase BirA indirectly repressed the LEE through a global regulator called Fur. This repression was characterized *in vitro* and was induced by biotin concentrations lower than that found in the small intestine but higher than that of the large intestine, consistent with their proposal. Furthermore, mice fed with a biotin rich diet during EHEC infection significantly reduced the amount of EHEC associated colonization when compared to mice maintained on a normal diet. This study revealed novel insights into LEE regulation by a physiologically relevant signal but also suggests the potential for modulating diet with a view to limiting EHEC infections. The effects were also found to be EHEC specific with EPEC not being responsive to biotin levels through this regulation (Yang et al., 2015). This further highlights the adaptive nature of LEE regulation in a pathotype specific manner.

## D-serine as a Niche Determinant of Pathogenic *E. coli*

D-amino acids have classically been seen as integral members of the bacterial peptidoglycan layer, with other roles being obscure due to the nature of D-amino acids not being utilized by bacterial ribosomes. Recently, studies have revealed that non-canonical D-amino acids are actively produced by diverse bacterial species and are used to regulate cell wall composition during different stages of growth (Lam et al., 2009; Cava et al., 2011). D-serine is a niche specific metabolite that is abundant in extra-intestinal sites of the human body, such as the urinary tract and the brain (Anfora et al., 2007; Wolosker et al., 2008). Indeed, D-serine acts as a neurotransmitter in the human brain but the high concentrations in urine are correlated to endogenous and exogenous sources (Nagata et al., 2007). For instance, processed foods are known to be concentrated in the d-isomeric form of amino acids (Man and Bada, 1987).

Owing to its abundance in the urinary tract. D-serine acts as a positive fitness trait and regulator of virulence genes in urinary pathogenic bacteria such as uropathogenic *E. coli* and *Staphylococcus saprophyticus* (Roesch et al., 2003; Anfora et al., 2007; Haugen et al., 2007; Sakinç et al., 2009; Korte-Berwanger et al., 2013). In contrast, EHEC cannot use D-serine as a carbon source due to a genetic replacement of the D-serine tolerance locus (Moritz and Welch, 2006). This replacement is widespread among the *E. coli* phylogeny and is almost always correlated with LEE-positive pathogens even though normal gut commensals can retain this locus. Analysis of the genome sequence of 1591 *E. coli* isolates revealed that carriage of both the LEE and the D-serine tolerance locus is an extremely rare event occurring in only 1.6% of cases. In accordance with this it was found that D-serine downregulates the LEE through indirect modulation of pre-existing transcriptional regulators Ihf and YhiF. The effect on LEE expression was also found to be independent of the ability to metabolize D-serine, explaining the lack of necessity to retain the D-serine tolerance locus in EHEC and separating metabolism from virulence. Uropathogenic *E. coli* do not encode the LEE T3SS and it was therefore proposed that the inhibitory action of D-serine was used by EHEC as an environmental signal to limit these pathogens to the intestine, an environment lacking an abundance of D-serine, thus reducing competition with extraintestinal *E. coli* pathogens (Connolly et al., 2015). This study further highlighted the impact that host metabolism can have on niche specification of bacterial pathogens.

## The Impact of the Microbiome on EHEC Infection

Enterohaemorrhagic *Escherichia coli* must actively compete with the resident microbiota in order to establish colonization of the host (Kamada et al., 2012). EHEC must penetrate the intestinal lumen in order to reach its preferred colonization site at the epithelial surface. This “zone of clearance” between the lumen and the cell surface is largely devoid of resident bacteria and the localized gluconeogenic conditions here promote LEE expression (Miranda et al., 2004; Vaishnava et al., 2011; Kamada et al., 2012). The microbiome is composed of trillions of bacteria from hundreds of species that have evolved to form a synergistic relationship with the host (Eckburg et al., 2005; Gill et al., 2006). In a sense, this relationship provides the first barrier of defense against invading pathogens. There is a wealth of recent research that highlights how diet can influence the composition of the microbiota and this in turn can have strong implications on the susceptibility of an individual to enteric infection given the essential roles of the microbiota in host metabolism and immunity (Turnbaugh and Gordon, 2009; Kau et al., 2011; Wu et al., 2011).

The majority of research on EHEC virulence regulation to date has involved detailed mapping of transcriptional regulatory networks *in vitro* complimented with *in vivo* infection models. However, some recent studies have revealed fascinating insights into the roles of the microbiota in the infection process. A study investigating the protective effect of species belonging to the bacterial genus *Bifidobacteria* revealed concentrations of acetate that were significantly higher in mice associated with these strains than non-protective *Bifidobacteria*. Species of *Bifidobacteria* were previously implicated in a protective manner by probiotic inhibition of EHEC. The mechanism of this protection involves the production of acetate in a glucose-independent manner associated with the distil part of the colon. To complement these findings the researchers also found that mice fed with a diet rich in acetylated starch had a significantly increased concentration of acetate in their feces and strongly suggested that targeted modulation of the diet should be considered as a therapeutic strategy to limit EHEC infections (Fukuda et al., 2011, 2012).

As discussed above the presence of favorable sugar sources can enhance disease progression of EHEC. Consistent with this, Curtis et al. (2014a) demonstrated that *B. thetaiotaomicron*, a major constituent of the intestinal microbiota, is involved in enriching the environment with simple monosaccharide sugars capable of being readily utilized by *E. coli* strains. The group had previously determined the mechanistic basis behind this, activation of the sugar sensitive transcription factor Cra, which leads to increased transcription of the LEE (Njoroge et al., 2012). In this study however, they identified a specific gluconeogenic substrate of Cra, succinate, which was responsible for increased disease severity in mice infected with *C. rodentium*. In contrast, antibiotic treated mice devoid of *B. thetaiotaomicron* were less susceptible to severe infection strongly suggesting that naturally produced sugars enhance the outcome of infection (Curtis et al., 2014a).

## Exploiting Virulence Regulation in the Quest for Novel Therapeutic Strategies

Resistance of bacterial pathogens to antibiotics is no longer an emerging theme but is now considered a global health concern (Davies and Davies, 2010). In an effort to combat antibiotic resistance much emphasis has been placed on developing the concept of antivirulence therapy. This can be defined as the inhibition of non-essential virulence factors without being detrimental to normal cell growth (Rasko and Sperandio, 2010). Several classes of antivirulence compounds have been described thus far as and have been recently reviewed in detail (Beckham and Roe, 2014; Zambelloni et al., 2015). Targeting regulatory systems is a promising strategy to inhibit the activity of the virulence factors. For instance, an inhibitor compound of QseC, LED209, acts by allosterically interacting with this receptor thus blocking the QseC signaling cascade required for expression of multiple virulence factors. The compound also elicits promising broad spectrum activity against multiple pathogens that all utilize the QseC signaling system to regulate virulence (Rasko et al., 2008; Curtis et al., 2014b).

Specifically targeting the T3SS is well established in the field of antivirulence given the non-essential nature of this appendage. The SA class of inhibitor compounds have been extensively described and are efficient inhibitors of Type III secretion in EHEC (Tree et al., 2009; Wang et al., 2011). The mechanism of action of these inhibitors is unclear but a study outlining the transcriptional effects of EHEC treated with two different SA compounds under conditions that stimulate the LEE resulted in inhibition of type 3 secretion but an increase in motility. Given the known cross-regulation between these two systems one could postulate that the mechanism of inhibition is targeting the regulatory circuit of the LEE as opposed to the T3SS itself.

The LEE of *C. rodentium* confers the A/E phenotype upon this mouse-specific pathogen therefore allowing *C. rodentium* to be used as a model for EHEC infections. This is a unique pathogen however, and despite the core regulation of the LEE being under the control of similar mechanisms, such as Ler and GrlRA, *C. rodentium* specific regulation of the LEE has been extensively described (Deng et al., 2004). RegA is a *C. rodentium* specific AraC-like transcriptional regulator controlling gene expression on a global scale (Hart et al., 2008). Over 40 ORFS are under the control of RegA including GrlRA. The system is also highly responsive to bicarbonate ions, which are involved in intestinal homeostasis (Yang et al., 2008, 2009). Bicarbonate is secreted to neutralize stomach acid and this is in turn sensed by RegA to regulate gene expression appropriately (Kaunitz and Akiba, 2006). This control feeds into the LEE in a similar manner to the GAD system to correctly express the T3SS under favorable environmental conditions. As well as characterizing RegA transcriptional regulation in detail, researchers have also targeted RegA directly as a therapeutic strategy. The team characterized a compound, Regacin, that specifically inhibits DNA binding of RegA thus was capable of reducing LEE expression and protecting mice from *C. rodentium* infection (Yang et al., 2013). This study provides an excellent model for screening and elucidation of mechanisms involving antivirulence compounds while providing proof of concept that targeting regulation of the LEE directly is a viable option for new therapeutics.

Finally, exploitation of our knowledge of metabolism and how it regulates virulence provides yet another exciting route to explore. As discussed in this review, the LEE is responsive to numerous physiologically relevant signals including host or microbiota derived metabolites. This makes sense considering how important microbiome-associated homeostasis is to a healthy functioning gut. With this in mind it is important to emphasize the idea of dietary supplementation in combatting EHEC infection. As mentioned above, Yang et al. (2015) reduced EHEC infection in mice treated with a biotin enriched diet whereas Fukuda et al. (2011) successfully treated mice against EHEC infection using acetylated starch feed, mimicking the effects of protective members of the microbiota that naturally produce this metabolite. Supplementary zinc has also been used to limit diarrheal infection in children (Sazawal et al., 1995; Crane et al., 2007). Zinc was shown to indirectly reduce transcription of the LEE, and thus expression of the T3SS in EPEC, by a mechanism involving activation of envelope stress (Mellies et al., 2012). Zinc is also important for immune homeostasis in the host and has a very low toxicity suggesting that increased administration of supplementary zinc may be a useful strategy to limiting diarrheal infection associated with LEE-positive pathogens (Prasad, 2007; Bolick et al., 2014).

## Conclusion

Nearly two decades of research on the LEE and how it is regulated has resulted in extensive knowledge of the system it encodes, the effects that A/E lesions and effector translocation have on the host, as well as how bacterial regulatory networks adapt to control this acquired genetic element appropriately. EHEC, EPEC, and *C. rodentium* have integrated the LEE island into their chromosomes and are capable of controlling its expression intuitively to respond to the environment as they travel through the host digestive system. There are many barriers to overcome along this journey such as survival in extreme pH and competition with the microbiota for limited nutrients, all the while responding to niche specific signals in order to regulate colonization appropriately. The detailed knowledge of how systems such as the QS network and microbiota associated metabolism affect regulation of the LEE has mediated the emergence of novel ways of targeting this virulence factor. These “bottom-up” approaches involving antivirulence compounds is an exciting field but much work remains to be done. What are the mechanisms of action of such strategies? How do they affect the pathogen and the host? Will this relationship lead to selective pressure for compound resistance? We are beginning to unravel these questions and advancing technologies are allowing more progressive ways of studying transcriptional regulation. For instance, the majority of work carried out on virulence factor regulation to date has been performed *in vitro* but *in vivo* regulation is much less explored. The application of next generation technologies such as RNA-sequencing (RNA-seq) to *in vivo* studies is allowing infection-relevant transcriptomic networks to be studied, as described for the bacterial pathogens *Vibrio cholerae*, *Aggregatibacter actinomycetemcomitans*, and more recently *Yersinia pseudotuberculosis* persistent infection (Mandlik et al., 2011; Jorth et al., 2013; Avican et al., 2015). Host and pathogen whole transcriptome profiling using RNA-sequencing (dual RNA-seq) is also emerging as the next step in infection-relevant gene expression studies and has been successfully applied to uropathogenic *E. coli* infected mouse macrophage samples to provide a comprehensive view of host-pathogen transcriptional responses to infection (Westermann et al., 2012; Mavromatis et al., 2015). The knowledge we have gained on LEE regulation is significant and continued work in this area in combination with next generation techniques will reveal more about pathogen adaptation to the host, strain specific regulatory factors and shed more light on the intricate relationship between virulence and metabolism, which has the potential to lead to new therapeutic strategies such as dietary supplementation to limit bacterial infections.

### Conflict of Interest Statement

The authors declare that the research was conducted in the absence of any commercial or financial relationships that could be construed as a potential conflict of interest.

## References

[B1] Abe H. Miyahara A. Oshima T. Tashiro K. Ogura Y. Kuhara S. (2008). Global regulation by horizontally transferred regulators establishes the pathogenicity of *Escherichia coli*. DNA Res. 15, 13–23. 10.1093/dnares/dsm033 18222925 PMC2650629

[B2] Anfora A. T. Haugen B. J. Roesch P. Redford P. Welch R. A. (2007). Roles of serine accumulation and catabolism in the colonization of the murine urinary tract by *Escherichia coli* CFT073. Infect. Immun. 75, 5298–5304. 10.1128/IAI.00652-07 17785472 PMC2168303

[B3] Atlung T. Ingmer H. (1997). H-NS: a modulator of environmentally regulated gene expression. Mol. Microbiol. 24, 7–17. 10.1046/j.1365-2958.1997.3151679.x 9140961

[B4] Avican K. Fahlgren A. Huss M. Heroven A. K. Beckstette M. Dersch P. (2015). Reprogramming of yersinia from virulent to persistent mode revealed by complex *in vivo* rna-seq analysis. PLoS Pathog. 11:e1004600. 10.1371/journal.ppat.1004600 25590628 PMC4295882

[B5] Barba J. Bustamante V. H. Flores-Valdez M. A. Deng W. Finlay B. B. Puente J. L. (2005). A positive regulatory loop controls expression of the locus of enterocyte effacement-encoded regulators Ler and GrlA. J. Bacteriol. 187, 7918–7930. 10.1128/JB.187.23.7918-7930.2005 16291665 PMC1291265

[B6] Beckham K. S. H. Connolly J. P. R. Ritchie J. M. Wang D. Gawthorne J. A. Tahoun A. (2014). The Metabolic enzyme AdhE controls the virulence of *Escherichia coli* O157:H7. Mol. Microbiol. 93, 199–211. 10.1111/mmi.12651 24846743 PMC4249723

[B7] Beckham K. S. H. Roe A. J. (2014). From screen to target: insights and approaches for the development of anti-virulence compounds. Front. Cell. Infect. Microbiol. 4:139. 10.3389/fcimb.2014.00139 25325019 PMC4179734

[B8] Berdichevsky T. Friedberg D. Nadler C. Rokney A. Oppenheim A. Rosenshine I. (2005). Ler is a negative autoregulator of the LEE1 operon in enteropathogenic *Escherichia coli*. J. Bacteriol. 187, 349–357. 10.1128/JB.187.1.349-357.2005 15601719 PMC538822

[B9] Bertin Y. Deval C. de la Foye A. Masson L. Gannon V. Harel J. (2014). The gluconeogenesis pathway is involved in maintenance of enterohaemorrhagic *Escherichia coli* O157:H7 in bovine intestinal content. PLoS ONE 9:e98367. 10.1371/journal.pone.0098367 24887187 PMC4041753

[B10] Bertin Y. Girardeau J. P. Chaucheyras-Durand F. Lyan B. Pujos-Guillot E. Harel J. (2011). Enterohaemorrhagic *Escherichia coli* gains a competitive advantage by using ethanolamine as a nitrogen source in the bovine intestinal content. Environ. Microbiol. 13, 365–377. 10.1111/j.1462-2920.2010.02334.x 20849446

[B11] Bolick D. T. Kolling G. L. Moore J. H. de Oliveira L. A. Tung K. Philipson C. (2014). Zinc deficiency alters host response and pathogen virulence in a mouse model of enteroaggregative *Escherichia coli*-induced diarrhea. Gut Microbes 5, 618–627. 10.4161/19490976.2014.969642 25483331 PMC4615194

[B12] Branchu P. Matrat S. Vareille M. Garrivier A. Durand A. Crépin S. (2014). NsrR, GadE, and GadX interplay in repressing expression of the *Escherichia coli* O157:H7 LEE pathogenicity island in response to nitric oxide. PLoS Pathog. 10:e1003874. 10.1371/journal.ppat.1003874 24415940 PMC3887101

[B13] Burkinshaw B. J. Deng W. Lameignère E. Wasney G. A. Zhu H. Worrall L. J. (2015). Structural analysis of a specialized type III secretion system peptidoglycan-cleaving enzyme. J. Biol. Chem. 290, 10406–10417. 10.1074/jbc.M115.639013 25678709 PMC4400350

[B14] Büttner D. (2012). Protein export according to schedule: architecture, assembly, and regulation of type III secretion systems from plant- and animal-pathogenic bacteria. Microbiol. Mol. Biol. Rev. 76, 262–310. 10.1128/MMBR.05017-11 22688814 PMC3372255

[B15] Cava F. de Pedro M. A. Lam H. Davis B. M. Waldor M. K. (2011). Distinct pathways for modification of the bacterial cell wall by non-canonical D-amino acids. EMBO J. 30, 3442–3453. 10.1038/emboj.2011.246 21792174 PMC3160665

[B16] Chang D.-E. Smalley D. J. Tucker D. L. Leatham M. P. Norris W. E. Stevenson S. J. (2004). Carbon nutrition of *Escherichia coli* in the mouse intestine. Proc. Natl. Acad. Sci. U.S.A. 101, 7427–7432. 10.1073/pnas.0307888101 15123798 PMC409935

[B17] Cho B.-K. Barrett C. L. Knight E. M. Park Y. S. Palsson B. Ø. (2008). Genome-scale reconstruction of the Lrp regulatory network in *Escherichia coli*. Proc. Natl. Acad. Sci. U.S.A. 105, 19462–19467. 10.1073/pnas.0807227105 19052235 PMC2614783

[B18] Clarke M. B. Hughes D. T. Zhu C. Boedeker E. C. Sperandio V. (2006). The QseC sensor kinase: a bacterial adrenergic receptor. Proc. Natl. Acad. Sci. U.S.A. 103, 10420–10425. 10.1073/pnas.0604343103 16803956 PMC1482837

[B19] Clarke M. B. Sperandio V. (2005). Transcriptional regulation of flhDC by QseBC and sigma (FliA) in enterohaemorrhagic *Escherichia coli*. Mol. Microbiol. 57, 1734–1749. 10.1111/j.1365-2958.2005.04792.x 16135237

[B20] Collins J. W. Keeney K. M. Crepin V. F. Rathinam V. A. K. Fitzgerald K. A. Finlay B. B. (2014). *Citrobacter rodentium*: infection, inflammation and the microbiota. Nat. Rev. Microbiol. 12, 612–623. 10.1038/nrmicro3315 25088150

[B21] Connolly J. P. Goldstone R. J. Burgess K. Cogdell R. J. Beatson S. A. Vollmer W. (2015). The host metabolite D-serine contributes to bacterial niche specificity through gene selection. ISME J. 9, 1039–1051. 10.1038/ismej.2014.242 25526369 PMC4366372

[B22] Crane J. K. Naeher T. M. Shulgina I. Zhu C. Boedeker E. C. (2007). Effect of zinc in enteropathogenic *Escherichia coli* infection. Infect. Immun. 75, 5974–5984. 10.1128/IAI.00750-07 17875638 PMC2168358

[B23] Croxen M. A. Finlay B. B. (2010). Molecular mechanisms of *Escherichia coli* pathogenicity. Nat. Rev. Microbiol. 8, 26–38. 10.1038/nrmicro2265 19966814

[B24] Croxen M. A. Law R. J. Scholz R. Keeney K. M. Wlodarska M. Finlay B. B. (2013). Recent advances in understanding enteric pathogenic *Escherichia coli*. Clin. Microbiol. Rev. 26, 822–880. 10.1128/CMR.00022-13 24092857 PMC3811233

[B25] Cummings J. H. Macfarlane G. T. (1991). The control and consequences of bacterial fermentation in the human colon. J. Appl. Bacteriol. 70, 443–459. 10.1111/j.1365-2672.1991.tb02739.x 1938669

[B26] Curtis M. M. Hu Z. Klimko C. Narayanan S. Deberardinis R. Sperandio V. (2014a). The gut commensal bacteroides thetaiotaomicron exacerbates enteric infection through modification of the metabolic landscape. Cell Host Microbe 16, 759–769. 10.1016/j.chom.2014.11.005 25498343 PMC4269104

[B27] Curtis M. M. Russell R. Moreira C. G. Adebesin A. M. Wang C. Williams N. S. (2014b). QseC inhibitors as an antivirulence approach for Gram-negative pathogens. MBio 5, e02165. 10.1128/mBio.02165-14 25389178 PMC4235214

[B28] Dame R. T. Noom M. C. Wuite G. J. L. (2006). Bacterial chromatin organization by H-NS protein unravelled using dual DNA manipulation. Nature 444, 387–390. 10.1038/nature05283 17108966

[B29] Davies J. Davies D. (2010). Origins and evolution of antibiotic resistance. Microbiol. Mol. Biol. Rev. 74, 417–433. 10.1128/MMBR.00016-10 20805405 PMC2937522

[B30] De Biase D. Tramonti A. Bossa F. Visca P. (1999). The response to stationary-phase stress conditions in *Escherichia coli*: role and regulation of the glutamic acid decarboxylase system. Mol. Microbiol. 32, 1198–1211. 10383761 10.1046/j.1365-2958.1999.01430.x

[B31] Deng W. Puente L. Gruenheid S. Li Y. Vallance B. A. Va A. (2004). Dissecting virulence: systematic and functional analyses of a pathogenicity island. Proc. Natl. Acad. Sci. U.S.A. 101, 3597–3602. 10.1073/pnas.0400326101 14988506 PMC373508

[B32] Deng W. Yu H. B. de Hoog C. L. Stoynov N. Li Y. Foster L. J. (2012). Quantitative proteomic analysis of type III secretome of enteropathogenic *Escherichia coli* reveals an expanded effector repertoire for attaching/effacing bacterial pathogens. Mol. Cell. Proteomics 11, 692–709. 10.1074/mcp.M111.013672 22661456 PMC3434768

[B33] Dillon S. C. Dorman C. J. (2010). Bacterial nucleoid-associated proteins, nucleoid structure and gene expression. Nat. Rev. Microbiol. 8, 185–195. 10.1038/nrmicro2261 20140026

[B34] Dong T. Coombes B. K. Schellhorn H. E. (2009). Role of RpoS in the virulence of *Citrobacter rodentium*. Infect. Immun. 77, 501–507. 10.1128/IAI.00850-08 18981255 PMC2612282

[B35] Dong T. Schellhorn H. E. (2009). Global effect of RpoS on gene expression in pathogenic *Escherichia coli* O157:H7 strain EDL933. BMC Genomics 10:349. 10.1186/1471-2164-10-349 19650909 PMC2907692

[B36] Dorman C. J. (2007). H-NS, the genome sentinel. Nat. Rev. Microbiol. 5, 157–161. 10.1038/nrmicro1598 17191074

[B37] Eckburg P. B. Bik E. M. Bernstein C. N. Purdom E. Dethlefsen L. Sargent M. (2005). Diversity of the human intestinal microbial flora. Science 308, 1635–1638. 10.1126/science.1110591 15831718 PMC1395357

[B38] Elliott S. J. Sperandio V. Girón J. A. Mellies J. L. Wainwright L. Steven W. (2000). The locus of enterocyte effacement (LEE)-encoded regulator controls expression of both LEE- and non-LEE-encoded virulence factors in enteropathogenic and enterohemorrhagic *Escherichia coli*. Infect. Immun. 68, 6115–6126. 10.1128/IAI.68.11.6115-6126.2000 11035714 PMC97688

[B39] Elliott S. J. Wainwright L. A. Timothy K. Jarvis K. G. Deng Y. Lai L. (1998). The complete sequence of the locus of enterocyte effacement (LEE) from enteropathogenic *Escherichia*. Mol. Microbiol. 28, 1–4. 9593291 10.1046/j.1365-2958.1998.00783.x

[B40] Elliott S. J. Yu J. Kaper J. B. (1999). The cloned locus of enterocyte effacement from enterohemorrhagic *Escherichia coli* O157:H7 is unable to confer the attaching and effacing phenotype upon *E*. *coli K-*12. Infect. Immun. 67, 4260–4263. 10417201 10.1128/iai.67.8.4260-4263.1999 PMC96734

[B41] Foley E. O’Farrell P. H. (2003). Nitric oxide contributes to induction of innate immune responses to gram-negative bacteria in *Drosophila*. Genes Dev. 17, 115–125. 10.1101/gad.1018503 12514104 PMC195964

[B42] Frankel G. Phillips A. D. Rosenshine I. Dougan G. Kaper J. B. Knutton S. (1998). Enteropathogenic and enterohaemorrhagic *Escherichia coli*: more subversive elements. Mol. Microbiol. 30, 911–921. 9988469 10.1046/j.1365-2958.1998.01144.x

[B43] Friedberg D. Umanski T. Fang Y. Rosenshine I. (1999). Hierarchy in the expression of the locus of enterocyte effacement genes of enteropathogenic *Escherichia coli*. Mol. Microbiol. 34, 941–952. 10.1046/j.1365-2958.1999.01655.x 10594820

[B44] Fukuda S. Toh H. Hase K. Oshima K. Nakanishi Y. Yoshimura K. (2011). Bifidobacteria can protect from enteropathogenic infection through production of acetate. Nature 469, 543–547. 10.1038/nature09646 21270894

[B45] Fukuda S. Toh H. Taylor T. D. Ohno H. Hattori M. (2012). Acetate-producing bifidobacteria protect the host from enteropathogenic infection via carbohydrate transporters. Gut Microbes 3, 449–454. 10.4161/gmic.21214 22825494

[B46] Giangrossi M. Zattoni S. Tramonti A. De Biase D. Falconi M. (2005). Antagonistic role of H-NS and GadX in the regulation of the glutamate decarboxylase-dependent acid resistance system in *Escherichia coli*. J. Biol. Chem. 280, 21498–21505. 10.1074/jbc.M413255200 15795232

[B47] Gill S. R. Pop M. Deboy R. T. Eckburg P. B. Turnbaugh P. J. Samuel B. S. (2006). Metagenomic analysis of the human distal gut microbiome. Science 312, 1355–1359. 10.1126/science.1124234 16741115 PMC3027896

[B48] Goldberg M. D. Johnson M. Hinton J. C. D. Williams P. H. (2001). Role of the nucleoid-associated protein Fis in the regulation of virulence properties of enteropathogenic *Escherichia coli*. Mol. Microbiol. 41, 549–559. 10.1046/j.1365-2958.2001.02526.x 11532124

[B49] Gonyar L. A. Kendall M. M. (2014). Ethanolamine and choline promote expression of putative and characterized fimbriae in enterohemorrhagic *Escherichia coli* O157:H7. Infect. Immun. 82, 193–201. 10.1128/IAI.00980-13 24126525 PMC3911853

[B50] Grainger D. C. Hurd D. Goldberg M. D. Busby S. J. W. (2006). Association of nucleoid proteins with coding and non-coding segments of the *Escherichia coli* genome. Nucleic Acids Res. 34, 4642–4652. 10.1093/nar/gkl542 16963779 PMC1636352

[B51] Gruber C. C. Sperandio V. (2014). Posttranscriptional control of microbe-induced rearrangement of host cell actin. MBio 5, e01025–13. 10.1128/mBio.01025-13 24425733 PMC3903284

[B52] Gruber C. C. Sperandio V. (2015). Global analysis of posttranscriptional regulation by GlmY and GlmZ in enterohemorrhagic *Escherichia coli* O157:H7. Infect. Immun. 83, 1286–1295. 10.1128/IAI.02918-14 25605763 PMC4363437

[B53] Haack K. R. Robinson C. L. Miller K. J. Fowlkes J. W. Mellies J. L. (2003). Interaction of Ler at the LEE5 (tir) operon of enteropathogenic *Escherichia coli*. Infect. Immun. 71, 384–392. 10.1128/IAI.71.1.384-392.2003 12496188 PMC143144

[B54] Habdas B. J. Smart J. Kaper J. B. Sperandio V. (2010). The LysR-type transcriptional regulator QseD alters type three secretion in enterohemorrhagic *Escherichia coli* and motility in K-12 *Escherichia coli*. J. Bacteriol. 192, 3699–3712. 10.1128/JB.00382-10 20494990 PMC2897335

[B55] Hansen A.-M. Kaper J. B. (2009). Hfq affects the expression of the LEE pathogenicity island in enterohaemorrhagic *Escherichia coli*. Mol. Microbiol. 73, 446–465. 10.1111/j.1365-2958.2009.06781.x 19570135 PMC2770234

[B56] Hart E. Yang J. Tauschek M. Kelly M. Wakefield M. J. Frankel G. (2008). RegA, an AraC-like protein, is a global transcriptional regulator that controls virulence gene expression in *Citrobacter rodentium*. Infect. Immun. 76, 5247–5256. 10.1128/IAI.00770-08 18765720 PMC2573378

[B57] Haugen B. J. Pellett S. Redford P. Hamilton H. L. Roesch P. L. Welch R. A. (2007). *In vivo* gene expression analysis identifies genes required for enhanced colonization of the mouse urinary tract by uropathogenic *Escherichia coli* strain CFT073 dsdA. Infect. Immun. 75, 278–289. 10.1128/IAI.01319-06 17074858 PMC1828413

[B58] Hayashi T. Makino K. Ohnishi M. Kurokawa K. Ishii K. Yokoyama K. (2001). Complete genome sequence of enterohemorrhagic *Escherichia coli* O157:H7 and genomic comparison with a laboratory strain K-12. DNA Res. 8, 11–22. 10.1093/dnares/8.1.11 11258796

[B59] Holmes A. Lindestam Arlehamn C. S. Wang D. Mitchell T. J. Evans T. J. Roe A. J. (2012). Expression and regulation of the *Escherichia coli* O157:H7 effector proteins NleH1 and NleH2. PLoS ONE 7:e33408. 10.1371/journal.pone.0033408 22428045 PMC3299786

[B60] Honda N. Iyoda S. Yamamoto S. Terajima J. Watanabe H. (2009). LrhA positively controls the expression of the locus of enterocyte effacement genes in enterohemorrhagic *Escherichia coli* by differential regulation of their master regulators PchA and PchB. Mol. Microbiol. 74, 1393–1341. 10.1111/j.1365-2958.2009.06937.x 19889091

[B61] Hughes D. T. Sperandio V. (2008). Inter-kingdom signalling: communication between bacteria and their hosts. Nat. Rev. Microbiol. 6, 111–120. 10.1038/nrmicro1836 18197168 PMC2667375

[B62] Hughes D. T. Terekhova D. A. Liou L. Hovde C. J. Sahl J. W. Patankar A. V. (2010). Chemical sensing in mammalian host-bacterial commensal associations. Proc. Natl. Acad. Sci. U.S.A. 107, 9831–9836. 10.1073/pnas.1002551107 20457895 PMC2906910

[B63] Ide T. Laarmann S. Greune L. Schillers H. Oberleithner H. Schmidt M. A. (2001). Characterization of translocation pores inserted into plasma membranes by type III-secreted Esp proteins of enteropathogenic *Escherichia coli*. Cell. Microbiol. 3, 669–679. 10.1046/j.1462-5822.2001.00146.x 11580752

[B64] Islam M. S. Bingle L. E. H. Pallen M. J. Busby S. J. W. (2011a). Organization of the LEE1 operon regulatory region of enterohaemorrhagic *Escherichia coli* O157:H7 and activation by GrlA. Mol. Microbiol. 79, 468–483. 10.1111/j.1365-2958.2010.07460.x 21219464

[B65] Islam M. S. Pallen M. J. Busby S. J. W. (2011b). A cryptic promoter in the LEE1 regulatory region of enterohaemorrhagic *Escherichia coli*: promoter specificity in AT-rich gene regulatory regions. Biochem. J. 436, 681–686. 10.1042/BJ20110260 21476984

[B66] Islam M. S. Shaw R. K. Frankel G. Pallen M. J. Busby S. J. W. (2012). Translation of a minigene in the 5^′^ leader sequence of the enterohaemorrhagic *Escherichia coli* LEE1 transcription unit affects expression of the neighbouring downstream gene. Biochem. J. 441, 247–253. 10.1042/BJ20110912 21973189 PMC3262186

[B67] Iyoda S. Koizumi N. Satou H. Lu Y. Saitoh T. Ohnishi M. (2006). The GrlR-GrlA regulatory system coordinately controls the expression of flagellar and LEE-encoded type III protein secretion systems in enterohemorrhagic *Escherichia coli*. J. Bacteriol. 188, 5682–5692. 10.1128/JB.00352-06 16885436 PMC1540053

[B68] Iyoda S. Watanabe H. (2004). Positive effects of multiple pch genes on expression of the locus of enterocyte effacement genes and adherence of enterohaemorrhagic *Escherichia coli* O157: H7 to HEp-2 cells. Microbiology 150, 2357–2571. 10.1099/mic.0.27100-0 15256577

[B69] Iyoda S. Watanabe H. (2005). ClpXP protease controls expression of the type III protein secretion system through regulation of RpoS and GrlR levels in enterohemorrhagic *Escherichia coli*. J. Bacteriol. 187, 4086–4094. 10.1128/JB.187.12.4086-4094.2005 15937171 PMC1151716

[B70] Jorth P. Trivedi U. Rumbaugh K. Whiteley M. (2013). Probing bacterial metabolism during infection using high-resolution transcriptomics. J. Bacteriol. 195, 4991–4998. 10.1128/JB.00875-13 23974023 PMC3811578

[B71] Kamada N. Kim Y.-G. Sham H. P. Vallance B. A. Puente J. L. Martens E. C. (2012). Regulated virulence controls the ability of a pathogen to compete with the gut microbiota. Science 336, 1325–1329. 10.1126/science.1222195 22582016 PMC3439148

[B72] Kanamaru K. Tatsuno I. Tobe T. Sasakawa C. (2000). SdiA, an *Escherichia coli* homologue of quorum-sensing regulators, controls the expression of virulence factors in enterohaemorrhagic *Escherichia coli* O157:H7. Mol. Microbiol. 38, 805–816. 10.1046/j.1365-2958.2000.02171.x 11115115

[B73] Karmali M. A. Petric M. Lim C. Fleming P. C. Steele B. T. (1983a). *Escherichia coli* cytotoxin, haemolytic-uraemic syndrome, and haemorrhagic colitis. Lancet 2, 1299–1300. 6139632 10.1016/s0140-6736(83)91167-4

[B74] Karmali M. A. Steele B. T. Petric M. Lim C. (1983b). Sporadic cases of haemolytic-uraemic syndrome associated with faecal cytotoxin and cytotoxin-producing *Escherichia coli* in stools. Lancet 1, 619–620. 6131302 10.1016/s0140-6736(83)91795-6

[B75] Kau A. L. Ahern P. P. Griffin N. W. Goodman A. L. Gordon J. I. (2011). Human nutrition, the gut microbiome and the immune system. Nature 474, 327–336. 10.1038/nature10213 21677749 PMC3298082

[B76] Kaunitz J. D. Akiba Y. (2006). Review article: duodenal bicarbonate—mucosal protection, luminal chemosensing and acid-base balance. Aliment. Pharmacol. Ther. 24(Suppl. 4), 169–176. 10.1111/j.1365-2036.2006.00041.x 17209861

[B77] Kendall M. M. Gruber C. C. Parker C. T. Sperandio V. (2012). Ethanolamine controls expression of genes encoding components involved in interkingdom signaling and virulence in enterohemorrhagic *Escherichia coli* O157:H7. MBio 3, e00050–12. 10.1128/mBio.00050-12 22589288 PMC3372972

[B78] Kendall M. M. Gruber C. C. Rasko D. A. Hughes D. T. Sperandio V. (2011). Hfq virulence regulation in enterohemorrhagic *Escherichia coli* O157:H7 strain 86-24. J. Bacteriol. 193, 6843–6851. 10.1128/JB.06141-11 21984790 PMC3232842

[B79] Kendall M. M. Rasko D. A. Sperandio V. (2010). The LysR-type regulator QseA regulates both characterized and putative virulence genes in enterohaemorrhagic *Escherichia coli* O157:H7. Mol. Microbiol. 76, 1306–1321. 10.1111/j.1365-2958.2010.07174.x 20444105 PMC2936457

[B80] Kenny B. Abe A. Stein M. Finlay B. B. (1997a). Enteropathogenic *Escherichia coli* protein secretion is induced in response to conditions similar to those in the gastrointestinal tract. Infect. Immun. 65, 2606–2612. 9199427 10.1128/iai.65.7.2606-2612.1997 PMC175369

[B81] Kenny B. DeVinney R. Stein M. Reinscheid D. J. Frey E. A. Finlay B. B. (1997b). Enteropathogenic *E. coli* (EPEC) transfers its receptor for intimate adherence into mammalian cells. Cell 91, 511–520. 9390560 10.1016/s0092-8674(00)80437-7

[B82] Kitagawa R. Takaya A. Yamamoto T. (2011). Dual regulatory pathways of flagellar gene expression by ClpXP protease in enterohaemorrhagic *Escherichia coli*. Microbiology 157, 3094–3103. 10.1099/mic.0.051151-0 21903756

[B83] Knutton S. Rosenshine I. Pallen M. J. Nisan I. Neves B. C. Bain C. (1998). A novel EspA-associated surface organelle of enteropathogenic *Escherichia coli* involved in protein translocation into epithelial cells. EMBO J. 17, 2166–2176. 10.1093/emboj/17.8.2166 9545230 PMC1170561

[B84] Korte-Berwanger M. Sakinc T. Kline K. Nielsen H. V Hultgren S. Gatermann S. G. (2013). Significance of the D-serine-deaminase and D-serine metabolism of Staphylococcus saprophyticus for virulence. Infect. Immun. 81, 4525–4533. 10.1128/IAI.00599-13 24082071 PMC3837983

[B85] Laaberki M.-H. Janabi N. Oswald E. Repoila F. (2006). Concert of regulators to switch on LEE expression in enterohemorrhagic *Escherichia coli* O157:H7: interplay between Ler, GrlA, HNS and RpoS. Int. J. Med. Microbiol. 296, 197–210. 10.1016/j.ijmm.2006.02.017 16618552

[B86] Lam H. Oh D.-C. Cava F. Takacs C. N. Clardy J. de Pedro M. A. (2009). D-amino acids govern stationary phase cell wall remodeling in bacteria. Science 325, 1552–1555. 10.1126/science.1178123 19762646 PMC2759711

[B87] Lathem W. W. Grys T. E. Witowski S. E. Torres A. G. Kaper J. B. Tarr P. I. (2002). StcE, a metalloprotease secreted by *Escherichia coli* O157:H7, specifically cleaves C1 esterase inhibitor. Mol. Microbiol. 45, 277–288. 10.1046/j.1365-2958.2002.02997.x 12123444

[B88] Leatham-Jensen M. P. Frimodt-Møller J. Adediran J. Mokszycki M. E. Banner M. E. Caughron J. E. (2012). The streptomycin-treated mouse intestine selects *Escherichia coli* envZ missense mutants that interact with dense and diverse intestinal microbiota. Infect. Immun. 80, 1716–1727. 10.1128/IAI.06193-11 22392928 PMC3347456

[B89] Lehnen D. Blumer C. Polen T. Wackwitz B. Wendisch V. F. Unden G. (2002). LrhA as a new transcriptional key regulator of flagella, motility and chemotaxis genes in *Escherichia coli*. Mol. Microbiol. 45, 521–532. 10.1046/j.1365-2958.2002.03032.x 12123461

[B90] Lodato P. B. Kaper J. B. (2009). Post-transcriptional processing of the LEE4 operon in enterohaemorrhagic *Escherichia coli*. Mol. Microbiol. 71, 273–290. 10.1111/j.1365-2958.2008.06530.x 19019141 PMC2782684

[B91] Luzader D. H. Clark D. E. Gonyar L. A. Kendall M. M. (2013). EutR is a direct regulator of genes that contribute to metabolism and virulence in enterohemorrhagic *Escherichia coli* O157:H7. J. Bacteriol. 195, 4947–4953. 10.1128/JB.00937-13 23995630 PMC3807496

[B92] Madrid C. Balsalobre C. García J. Juárez A. (2007). The novel Hha/YmoA family of nucleoid-associated proteins: use of structural mimicry to modulate the activity of the H-NS family of proteins. Mol. Microbiol. 63, 7–14. 10.1111/j.1365-2958.2006.05497.x 17116239

[B93] Mahajan A. Currie C. G. Mackie S. Tree J. McAteer S. McKendrick I. (2009). An investigation of the expression and adhesin function of H7 flagella in the interaction of *Escherichia coli* O157:H7 with bovine intestinal epithelium. Cell. Microbiol. 11, 121–137. 10.1111/j.1462-5822.2008.01244.x 19016776

[B94] Man E. H. Bada J. L. (1987). Dietary D-amino acids. Annu. Rev. Nutr. 7, 209–225. 10.1146/annurev.nu.07.070187.001233 3300733

[B95] Mandlik A. Livny J. Robins W. P. Ritchie J. M. Mekalanos J. J. Waldor M. K. (2011). RNA-Seq-based monitoring of infection-linked changes in Vibrio cholerae gene expression. Cell Host Microbe 10, 165–174. 10.1016/j.chom.2011.07.007 21843873 PMC3166260

[B96] Masuda N. Church G. M. (2003). Regulatory network of acid resistance genes in *Escherichia coli*. Mol. Microbiol. 48, 699–712. 10.1046/j.1365-2958.2003.03477.x 12694615

[B97] Mavromatis C. H. Bokil N. J. Totsika M. Kakkanat A. Schaale K. Cannistraci C. V. (2015). The co-transcriptome of uropathogenic *Escherichia coli*-infected mouse macrophages reveals new insights into host-pathogen interactions. Cell. Microbiol. 17, 730–746. 10.1111/cmi.12397 25410299 PMC4950338

[B98] McDaniel T. K. Jarvis K. G. Donnenberg M. S. Kaper J. B. (1995). A genetic locus of enterocyte effacement conserved among diverse enterobacterial pathogens. Proc. Natl. Acad. Sci. U.S.A. 92, 1664–1668. 10.1073/pnas.92.5.1664 7878036 PMC42580

[B99] McDaniel T. K. Kaper J. B. (1997). A cloned pathogenicity island from enteropathogenic *Escherichia coli* confers the attaching and effacing phenotype on E. *coli K-*12. Mol. Microbiol. 23, 399–407. 9044273 10.1046/j.1365-2958.1997.2311591.x

[B100] Meador J. P. Caldwell M. E. Cohen P. S. Conway T. (2014). *Escherichia coli* pathotypes occupy distinct niches in the mouse intestine. Infect. Immun. 82, 1931–1938. 10.1128/IAI.01435-13 24566621 PMC3993424

[B101] Mellies J. L. Barron A. M. S. Carmona A. M. (2007). Enteropathogenic and enterohemorrhagic *Escherichia coli* virulence gene regulation. Infect. Immun. 75, 4199–4210. 10.1128/IAI.01927-06 17576759 PMC1951183

[B102] Mellies J. L. Elliott S. J. Sperandio V. Donnenberg M. S. Kaper J. B. (1999). The Per regulon of enteropathogenic *Escherichia coli*: identification of a regulatory cascade and a novel transcriptional activator, the locus of enterocyte effacement (LEE)-encoded regulator (Ler). Mol. Microbiol. 33, 296–306. 10411746 10.1046/j.1365-2958.1999.01473.x

[B103] Mellies J. L. Thomas K. Turvey M. Evans N. R. Crane J. Boedeker E. (2012). Zinc-induced envelope stress diminishes type III secretion in enteropathogenic *Escherichia coli*. BMC Microbiol. 12:123. 10.1186/1471-2180-12-123 22727253 PMC3438133

[B104] Miranda R. L. Conway T. Leatham M. P. Chang D. E. Norris W. E. Allen J. H. (2004). Glycolytic and gluconeogenic growth of *Escherichia coli* O157:H7 (EDL933) and *E*. *coli K-*12 (MG1655) in the mouse intestine. Infect. Immun. 72, 1666–1676. 10.1128/IAI.72.3.1666-1676.2004 14977974 PMC355998

[B105] Moon H. W. Whipp S. C. Argenzio R. A. Levine M. M. Giannella R. A. (1983). Attaching and effacing activities of rabbit and human enteropathogenic *Escherichia coli* in pig and rabbit intestines. Infect. Immun. 41, 1340–1351. 6350186 10.1128/iai.41.3.1340-1351.1983 PMC264644

[B106] Moritz R. L. Welch R. A. (2006). The *Escherichia coli* argW-dsdCXA genetic island is highly variable, and *E. coli K*1 strains commonly possess two copies of dsdCXA. J. Clin. Microbiol. 44, 4038–4048. 10.1128/JCM.01172-06 17088369 PMC1698345

[B107] Nagata Y. Sato T. Enomoto N. Ishii Y. Sasaki K. Yamada T. (2007). High concentrations of D-amino acids in human gastric juice. Amino Acids 32, 137–140. 10.1007/s00726-006-0262-9 16583309

[B108] Nakanishi N. Tashiro K. Kuhara S. Hayashi T. Sugimoto N. Tobe T. (2009). Regulation of virulence by butyrate sensing in enterohaemorrhagic *Escherichia coli*. Microbiology 155, 521–530. 10.1099/mic.0.023499-0 19202100

[B109] Nataro J. P. Kaper J. B. (1998). Diarrheagenic *Escherichia coli*. Clin. Microbiol. Rev. 11, 142–201. 9457432 10.1128/cmr.11.1.142 PMC121379

[B110] Navarre W. W. Porwollik S. Wang Y. McClelland M. Rosen H. Libby S. J. (2006). Selective silencing of foreign DNA with low GC content by the H-NS protein in *Salmonella*. Science 313, 236–238. 10.1126/science.1128794 16763111

[B111] Naylor S. W. Low J. C. Besser T. E. Mahajan A. Gunn G. J. Pearce M. C. (2003). Lymphoid follicle-dense mucosa at the terminal rectum is the principal site of colonization of enterohemorrhagic *Escherichia coli* O157:H7 in the bovine host. Infect. Immun. 71, 1505–1512. 10.1128/IAI.71.3.1505-1512.2003 12595469 PMC148874

[B112] Nguyen Y. N. Sheng H. Dakarapu R. Falck J. R. Hovde C. J. Sperandio V. (2013). The acyl-homoserine lactone synthase YenI from *Yersinia enterocolitica* modulates virulence gene expression in enterohemorrhagic *Escherichia coli* O157:H7. Infect. Immun. 81, 4192–4199. 10.1128/IAI.00889-13 23980115 PMC3811827

[B113] Njoroge J. W. Nguyen Y. Curtis M. M. Moreira C. G. Sperandio V. (2012). Virulence meets metabolism: Cra and KdpE gene regulation in enterohemorrhagic *Escherichia coli*. MBio 3, e00280–12. 10.1128/mBio.00280-12 23073764 PMC3482499

[B114] Orskov F. Orskov I. Villar J. A. (1987). Cattle as reservoir of verotoxin-producing *Escherichia coli* O157:H7. Lancet 2, 276. 2886741 10.1016/s0140-6736(87)90860-9

[B115] Oshima T. Ishikawa S. Kurokawa K. Aiba H. Ogasawara N. (2006). *Escherichia coli* histone-like protein H-NS preferentially binds to horizontally acquired DNA in association with RNA polymerase. DNA Res. 13, 141–153. 10.1093/dnares/dsl009 17046956

[B116] Pacheco A. R. Curtis M. M. Ritchie J. M. Munera D. Waldor M. K. Moreira C. G. (2012). Fucose sensing regulates bacterial intestinal colonization. Nature 492, 113–117. 10.1038/nature11623 23160491 PMC3518558

[B117] Padavannil A. Jobichen C. Mills E. Velazquez-Campoy A. Li M. Leung K. Y. (2013). Structure of GrlR-GrlA complex that prevents GrlA activation of virulence genes. Nat. Commun. 4, 2546. 10.1038/ncomms3546 24092262

[B118] Perna N. T. Plunkett G. Burland V. Mau B. Glasner J. D. Rose D. J. (2001). Genome sequence of enterohaemorrhagic *Escherichia coli* O157:H7. Nature 409, 529–533. 10.1038/35054089 11206551

[B119] Prasad A. S. (2007). Zinc: mechanisms of host defense. J. Nutr. 137, 1345–1349. 17449604 10.1093/jn/137.5.1345

[B120] Prieto A. I. Kahramanoglou C. Ali R. M. Fraser G. M. Seshasayee A. S. N. Luscombe N. M. (2012). Genomic analysis of DNA binding and gene regulation by homologous nucleoid-associated proteins IHF and HU in *Escherichia coli* K12. Nucleic Acids Res. 40, 3524–3537. 10.1093/nar/gkr1236 22180530 PMC3333857

[B121] Pruimboom-Brees I. M. Morgan T. W. Ackermann M. R. Nystrom E. D. Samuel J. E. Cornick N. A. (2000). Cattle lack vascular receptors for *Escherichia coli* O157:H7 Shiga toxins. Proc. Natl. Acad. Sci. U.S.A. 97, 10325–10329. 10.1073/pnas.190329997 10973498 PMC27023

[B122] Rasko D. A. Moreira C. G. Li D. R. Reading N. C. Ritchie J. M. Waldor M. K. (2008). Targeting QseC signaling and virulence for antibiotic development. Science 321, 1078–1080. 10.1126/science.1160354 18719281 PMC2605406

[B123] Rasko D. A. Sperandio V. (2010). Anti-virulence strategies to combat bacteria-mediated disease. Nat. Rev. Drug Discov. 9, 117–128. 10.1038/nrd3013 20081869

[B124] Reading N. C. Rasko D. A. Torres A. G. Sperandio V. (2009). The two-component system QseEF and the membrane protein QseG link adrenergic and stress sensing to bacterial pathogenesis. Proc. Natl. Acad. Sci. U.S.A. 106, 5889–5894. 10.1073/pnas.0811409106 19289831 PMC2667056

[B125] Reading N. C. Torres A. G. Kendall M. M. Hughes D. T. Yamamoto K. Sperandio V. (2007). A novel two-component signaling system that activates transcription of an enterohemorrhagic *Escherichia coli* effector involved in remodeling of host actin. J. Bacteriol. 189, 2468–2476. 10.1128/JB.01848-06 17220220 PMC1899401

[B126] Ren C.-P. Chaudhuri R. R. Fivian A. Bailey C. M. Antonio M. Barnes W. M. (2004). The ETT2 gene cluster, encoding a second type III secretion system from *Escherichia coli*, is present in the majority of strains but has undergone widespread mutational attrition. J. Bacteriol. 186, 3547–3560. 10.1128/JB.186.11.3547-3560.2004 15150243 PMC415751

[B127] Riley L. W. Remis R. S. Helgerson S. D. McGee H. B. Wells J. G. Davis B. R. (1983). Hemorrhagic colitis associated with a rare *Escherichia coli* serotype. N. Engl. J. Med. 308, 681–685. 10.1056/NEJM198303243081203 6338386

[B128] Roe A. J. Tysall L. Dransfield T. Wang D. Fraser-Pitt D. Mahajan A. (2007). Analysis of the expression, regulation and export of NleA-E in *Escherichia coli* O157:H7. Microbiology 153, 1350–1360. 10.1099/mic.0.2006/003707-0 17464049

[B129] Roe A. J. Yull H. Naylor S. W. Martin J. Smith D. G. E. Gally D. L. (2003). Heterogeneous surface expression of EspA translocon filaments by *Escherichia coli* O157:H7 is controlled at the posttranscriptional level. Infect. Immun. 71, 5900–5909. 10.1128/IAI.71.10.5900-5909.2003 14500511 PMC201059

[B130] Roesch P. L. Redford P. Batchelet S. Moritz R. L. Pellett S. Haugen B. J. (2003). Uropathogenic *Escherichia coli* use D-serine deaminase to modulate infection of the murine urinary tract. Mol. Microbiol. 49, 55–67. 10.1046/j.1365-2958.2003.03543.x 12823810

[B131] Rosenshine I. Ruschkowski S. Finlay B. B. (1996). Expression of attaching/effacing activity by enteropathogenic *Escherichia coli* depends on growth phase, temperature, and protein synthesis upon contact with epithelial cells. Infect. Immun. 64, 966–973. 8641808 10.1128/iai.64.3.966-973.1996 PMC173864

[B132] Russell R. M. Sharp F. C. Rasko D. A. Sperandio V. (2007). QseA and GrlR/GrlA regulation of the locus of enterocyte effacement genes in enterohemorrhagic *Escherichia coli*. J. Bacteriol. 189, 5387–5392. 10.1128/JB.00553-07 17496094 PMC1951852

[B133] Sakinç T. Michalski N. Kleine B. Gatermann S. G. (2009). The uropathogenic species Staphylococcus saprophyticus tolerates a high concentration of D-serine. FEMS Microbiol. Lett. 299, 60–64. 10.1111/j.1574-6968.2009.01731.x 19674114

[B134] Sánchez-SanMartín C. Bustamante V. H. Calva E. Puente J. L. (2001). Transcriptional regulation of the orf19 gene and the tir-cesT-eae operon of enteropathogenic *Escherichia coli*. J. Bacteriol. 183, 2823–2833. 10.1128/JB.183.9.2823-2833.2001 11292802 PMC99499

[B135] Sazawal S. Black R. E. Bhan M. K. Bhandari N. Sinha A. Jalla S. (1995). Zinc supplementation in young children with acute diarrhea in India. N. Engl. J. Med. 333, 839–844. 10.1056/NEJM199509283331304 7651474

[B136] Schauer D. B. Falkow S. (1993). Attaching and effacing locus of a *Citrobacter freundii* biotype that causes transmissible murine colonic hyperplasia. Infect. Immun. 61, 2486–2492. 8500884 10.1128/iai.61.6.2486-2492.1993 PMC280873

[B137] Shakhnovich E. A. Davis B. M. Waldor M. K. (2009). Hfq negatively regulates type III secretion in EHEC and several other pathogens. Mol. Microbiol. 74, 347–363. 10.1111/j.1365-2958.2009.06856.x 19703108 PMC2765575

[B138] Sharma V. K. Zuerner R. L. (2004). Role of hha and ler in transcriptional regulation of the esp operon of enterohemorrhagic *Escherichia coli* O157:H7. J. Bacteriol. 186, 7290–7301. 10.1128/JB.186.21.7290-7301.2004 15489441 PMC523200

[B139] Sharp F. C. Sperandio V. (2007). QseA directly activates transcription of LEE1 in enterohemorrhagic *Escherichia coli*. Infect. Immun. 75, 2432–2440. 10.1128/IAI.02003-06 17339361 PMC1865749

[B140] Shin S. Castanie-Cornet M. P. Foster J. W. Crawford J. A. Brinkley C. Kaper J. B. (2001). An activator of glutamate decarboxylase genes regulates the expression of enteropathogenic *Escherichia coli* virulence genes through control of the plasmid-encoded regulator, Per. Mol. Microbiol. 41, 1133–1150. 10.1046/j.1365-2958.2001.02570.x 11555293

[B141] Sperandio V. Li C. C. Kaper J. B. (2002a). Quorum-sensing *Escherichia coli* regulator A: a regulator of the LysR family involved in the regulation of the locus of enterocyte effacement pathogenicity island in enterohemorrhagic *E. coli*. Infect. Immun. 70, 3085–3093. 10.1128/IAI.70.6.3085-3093.2002 12011002 PMC127966

[B142] Sperandio V. Torres A. G. Kaper J. B. (2002b). Quorum sensing *Escherichia coli* regulators B and C (QseBC): a novel two-component regulatory system involved in the regulation of flagella and motility by quorum sensing in *E. coli*. Mol. Microbiol. 43, 809–821. 10.1046/j.1365-2958.2002.02803.x 11929534

[B143] Sperandio V. Mellies J. L. Nguyen W. Shin S. Kaper J. B. (1999). Quorum sensing controls expression of the type III secretion gene transcription and protein secretion in enterohemorrhagic and enteropathogenic *Escherichia coli*. Proc. Natl. Acad. Sci. U.S.A. 96, 15196–15201. 10611361 10.1073/pnas.96.26.15196 PMC24796

[B144] Takao M. Yen H. Tobe T. (2014). LeuO enhances butyrate-induced virulence expression through a positive regulatory loop in enterohaemorrhagic *Escherichia coli*. Mol. Microbiol. 93, 1302–1313. 10.1111/mmi.12737 25069663

[B145] Tatsuno I. Nagano K. Taguchi K. Rong L. Mori H. Sasakawa C. (2003). Increased adherence to Caco-2 cells caused by disruption of the yhiE and yhiF genes in enterohemorrhagic *Escherichia coli* O157:H7. Infect. Immun. 71, 2598–2606. 10.1128/IAI.71.5.2598-2606.2003 12704134 PMC153261

[B146] Tan A. Petty N. K. Hocking D. Bennett-Wood V. Wakefield M. Praszkier J. (2015). Evolutionary adaptation of an AraC-like regulatory protein in *Citrobacter rodentium* and *Escherichia* species. Infect. Immun. 83, 1384–1395. 10.1128/IAI.02697-14 25624355 PMC4363417

[B147] Tobe T. Beatson S. A. Taniguchi H. Abe H. Bailey C. M. Fivian A. (2006). An extensive repertoire of type III secretion effectors in *Escherichia coli* O157 and the role of lambdoid phages in their dissemination. Proc. Natl. Acad. Sci. U.S.A. 103, 14941–14946. 10.1073/pnas.0604891103 16990433 PMC1595455

[B148] Tobe T. Nakanishi N. Sugimoto N. (2011). Activation of motility by sensing short-chain fatty acids via two steps in a flagellar gene regulatory cascade in enterohemorrhagic *Escherichia coli*. Infect. Immun. 79, 1016–1024. 10.1128/IAI.00927-10 21149585 PMC3067497

[B149] Tree J. J. Roe A. J. Flockhart A. McAteer S. P. Xu X. Shaw D. (2011). Transcriptional regulators of the GAD acid stress island are carried by effector protein-encoding prophages and indirectly control type III secretion in enterohemorrhagic *Escherichia coli* O157:H7. Mol. Microbiol. 80, 1349–1365. 10.1111/j.1365-2958.2011.07650.x 21492263 PMC7099609

[B150] Tree J. J. Wang D. McInally C. Mahajan A. Layton A. Houghton I. (2009). Characterization of the effects of salicylidene acylhydrazide compounds on type III secretion in *Escherichia coli* O157:H7. Infect. Immun. 77, 4209–4220. 10.1128/IAI.00562-09 19635828 PMC2747932

[B151] Turnbaugh P. J. Gordon J. I. (2009). The core gut microbiome, energy balance and obesity. J. Physiol. 587, 4153–4158. 10.1113/jphysiol.2009.174136 19491241 PMC2754355

[B152] Umanski T. Rosenshine I. Friedberg D. (2002). Thermoregulated expression of virulence genes in enteropathogenic *Escherichia coli*. Microbiology 148, 2735–2744. 12213920 10.1099/00221287-148-9-2735

[B153] Vaishnava S. Yamamoto M. Severson K. M. Ruhn K. A. Yu X. Koren O. (2011). The antibacterial lectin RegIIIgamma promotes the spatial segregation of microbiota and host in the intestine. Science 334, 255–258. 10.1126/science.1209791 21998396 PMC3321924

[B154] Walters M. Sperandio V. (2006). Autoinducer 3 and epinephrine signaling in the kinetics of locus of enterocyte effacement gene expression in enterohemorrhagic *Escherichia coli*. Infect. Immun. 74, 5445–5455. 10.1128/IAI.00099-06 16988219 PMC1594898

[B155] Wang D. Zetterström C. E. Gabrielsen M. Beckham K. S. H. Tree J. J. Macdonald S. E. (2011). Identification of bacterial target proteins for the salicylidene acylhydrazide class of virulence-blocking compounds. J. Biol. Chem. 286, 29922–29931. 10.1074/jbc.M111.233858 21724850 PMC3191033

[B156] Westermann A. J. Gorski S. A. Vogel J. (2012). Dual RNA-seq of pathogen and host. Nat. Rev. Microbiol. 10, 618–630. 10.1038/nrmicro2852 22890146

[B157] Wolosker H. Dumin E. Balan L. Foltyn V. N. (2008). D-amino acids in the brain: D-serine in neurotransmission and neurodegeneration. FEBS J. 275, 3514–3526. 10.1111/j.1742-4658.2008.06515.x 18564180

[B158] Wong A. R. C. Pearson J. S. Bright M. D. Munera D. Robinson K. S. Lee S. F. (2011). Enteropathogenic and enterohaemorrhagic *Escherichia coli*: even more subversive elements. Mol. Microbiol. 80, 1420–1438. 10.1111/j.1365-2958.2011.07661.x 21488979

[B159] Wong J. M. W. de Souza R. Kendall C. W. C. Emam A. Jenkins D. J. A. (2006). Colonic health: fermentation and short chain fatty acids. J. Clin. Gastroenterol. 40, 235–243. 10.1097/00004836-200603000-00015 16633129

[B160] Wu G. D. Chen J. Hoffmann C. Bittinger K. Chen Y.-Y. Keilbaugh S. A. (2011). Linking long-term dietary patterns with gut microbial enterotypes. Science 334, 105–108. 10.1126/science.1208344 21885731 PMC3368382

[B161] Yang B. Feng L. Wang F. Wang L. (2015). Enterohemorrhagic *Escherichia coli* senses low biotin status in the large intestine for colonization and infection. Nat. Commun. 6, 6592. 10.1038/ncomms7592 25791315 PMC4382993

[B162] Yang J. Dogovski C. Hocking D. Tauschek M. Perugini M. Robins-Browne R. M. (2009). Bicarbonate-mediated stimulation of RegA, the global virulence regulator from *Citrobacter rodentium*. J. Mol. Biol. 394, 591–599. 10.1016/j.jmb.2009.10.033 19853617

[B163] Yang J. Hart E. Tauschek M. Price G. D. Hartland E. L. Strugnell R. A. (2008). Bicarbonate-mediated transcriptional activation of divergent operons by the virulence regulatory protein, RegA, from *Citrobacter rodentium*. Mol. Microbiol. 68, 314–327. 10.1111/j.1365-2958.2008.06171.x 18284589

[B164] Yang J. Hocking D. M. Cheng C. Dogovski C. Perugini M. A. Holien J. K. (2013). Disarming bacterial virulence through chemical inhibition of the DNA binding domain of an AraC-like transcriptional activator protein. J. Biol. Chem. 288, 31115–31126. 10.1074/jbc.M113.503912 24019519 PMC3829424

[B165] Yona-Nadler C. Umanski T. Aizawa S.-I. Friedberg D. Rosenshine I. (2003). Integration host factor (IHF) mediates repression of flagella in enteropathogenic and enterohaemorrhagic *Escherichia coli*. Microbiology 149, 877–884. 10.1099/mic.0.25970-0 12686630

[B166] Zambelloni R. Marquez R. Roe A. J. (2015). Development of antivirulence compounds: a biochemical review. Chem. Biol. Drug Des. 85, 43–55. 10.1111/cbdd.12430 25521644

[B167] Zhang L. Chaudhuri R. R. Constantinidou C. Hobman J. L. Patel M. D. Jones A. C. (2004). Regulators encoded in the *Escherichia coli* type III secretion system 2 gene cluster influence expression of genes within the locus for enterocyte effacement in enterohemorrhagic E. *coli O*157:H7. Infect. Immun. 72, 7282–7293. 10.1128/IAI.72.12.7282-7293.2004 15557654 PMC529121

